# Much Ado about N…atrium: modelling tissue sodium as a highly sensitive marker of subclinical and localized oedema

**DOI:** 10.1042/CS20180575

**Published:** 2018-12-13

**Authors:** Giacomo Rossitto, Rhian M. Touyz, Mark C. Petrie, Christian Delles

**Affiliations:** Institute of Cardiovascular and Medical Sciences, University of Glasgow, Glasgow, U.K.

**Keywords:** concentration, model, MRI, oedema, sodium

## Abstract

Hypertonic Na^+^ accumulation in peripheral tissues is a recently described phenomenon: it has been associated with ageing, hypertension, diabetes, chronic kidney disease and heart failure, but its clinical meaning has yet to be determined. This concept conflicts with the classic physiological paradigm of constant balance between salt intake and excretion, and its water-independent nature is still a matter of debate.

We developed a theoretical model explaining changes in the chemical composition of tissues as a function of extracellular volume fraction and excess extracellular fluid, i.e. oedema. The model suggests that the proportional increase in absolute Na^+^ content and concentration due to different degrees of oedema is higher than the parallel increase in water content, thus making Na^+^ a more sensitive index to detect this oedema.

Our model would explain some of the recent findings of high tissue Na^+^ content in pathological conditions. More importantly, it prompts the reappraisal of tissue Na^+^ analysis from being a topic of niche interest to a potential diagnostic tool with broad applicability in the investigation of subclinical systemic and localized oedema.

## A paradigm shift: from equilibrium to local hypertonic sodium accumulation

Classic physiology advocates the ultimate achievement of a constant balance between salt intake and excretion. At variance, over the last few years work by Titze and his collaborators has highlighted the phenomenon of hypertonic sodium (Na^+^) accumulation in peripheral tissues. Having observed considerable changes in total body Na^+^ without corresponding changes in body weight in a human long-term balance study [1], they first proposed skin as an extensive depot for water-independent Na^+^ storage based on studies in rodents [2]. Further preclinical investigations identified local regulatory mechanisms, entailing glycosaminoglycans as the putative binding site and tonicity-dependent modulation of skin lymphatic vessels as a buffering system [3,4], as well as the hypertensive phenotype associated with their disruption.

Availability of ^23^Na-magnetic resonance imaging (^23^Na-MRI) allowed translation back to humans: an increase in skin Na^+^ content occurs with ageing; in patients with refractory hypertension or primary aldosteronism compared to normotensive controls [5,6]; and in other clinical conditions such as acute heart failure [7], systemic sclerosis [8] and diabetes [9]. Skin Na^+^ also correlates better than total body overhydration or blood pressure with left ventricular mass in patients with chronic kidney disease [10], thus stimulating the quest for interventions specifically targeting tissue Na^+^ to improve cardiovascular outcomes in this population.

Overall, this new notion of local Na^+^ accumulation was acknowledged by independent influential reports as a paradigm shift [11,12], closely linked to the enormous public health burden of ageing and cardiovascular disease, which deserves a clearer understanding [12].

## A Na^+^ model beyond the skin

The above MRI studies, which were all performed in the lower leg, revealed an increase in ^23^Na signal not only in the skin but also in the skeletal muscle [6,7,9]; this is at odds with mechanisms for hypertonic Na^+^ accumulation, described as skin-specific in the preclinical studies.

In an attempt to study whether a similar phenomenon could be generalized to other tissues, we analyzed myocardial water and total electrolyte composition in a rodent model of ageing and hypertension [13]. After tissue digestion, we observed an increase in tissue Na^+^ content and concentration, which was not hypertonic, but paralleled by water accrual and an associated decrease in potassium (K^+^) concentration, previously described by others as a ‘loss’ [2].

**Figure 1:**
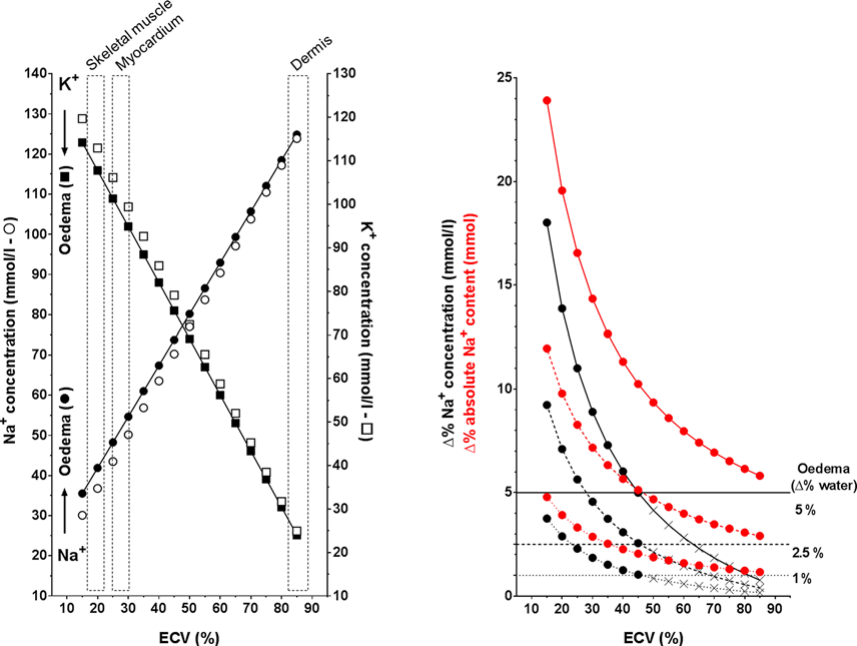
Model for chemical composition of tissues and impact of oedema Left panel: Expected total concentration of Na^+^ (open circles) and K^+^ (open squares) in a tissue, before and after addition of 5% of oedema (closed squares and circles, respectively), as a function of the extracellular volume fraction (ECV%). Values for representative tissues with different ECV% are shown in dashed boxes. Right panel: Percentage change of tissue Na^+^ concentration (black) or absolute content (red) after addition of 1% (dotted line), 2.5% (dashed line) or 5% (continuous line) oedema to the tissue, as a function of the extracellular volume fraction (ECV%).

We, therefore, developed a simple mathematical model (Supplementary Information for calculations) for predicting total Na^+^ and K^+^ composition of any tissue, as a function of the extracellular volume fraction (ECV%): intuitively, the higher the ECV% (i.e. the proportion of the Na^+^-rich extracellular solution), the higher the total concentration of Na^+^ in the ‘final solution’ from whole tissue (Figure 1, left panel, open circles). Vice versa, the decrease in K^+^ concentration is explained by a proportionally less represented intracellular, K^+^-rich compartment (Figure 1, left panel, open squares), without any ‘loss’.

More importantly, we interpreted the increase in water content and the overall unchanged sum of Na^+^ and K^+^ in our experiment as ‘isotonic’ oedema. Accordingly, we challenged the above model by simulating the effect of adding a fixed and biologically plausible moiety of fluid (e.g. 1%, 2.5% or 5%), equal in composition to the extracellular Na^+^-rich solution, to the tissues (i.e. oedema). This shifted the curves up for Na^+^ and down for K^+^, as expected from the equivalent of a global increase in ECV% (Figure 1, left panel, closed symbols for 5% oedema). Unexpectedly, however, we also noted that the proportional increase in tissue Na^+^ concentration due to oedema was much higher than the increase in water in tissues where the ECV% is low, such as skeletal muscle and myocardium with an ECV% of 16–20% and 25–30%, respectively (Figure 1, right panel; black lines). This phenomenon is even more pronounced and holds true across all the spectrum of possible ECV% when absolute Na^+^ content, rather than concentration, is taken into account (Figure 1, right panel; red lines).

With due consideration of experimental variability, this theoretical model is in keeping with data from us and others [2–4,13,14]. Moreover, the concept of a ‘final solution’ applies to chemical analysis of ashed [2,4] and/or digested [13] tissues, but also to any other technique that does not allow (accurate) spatial differentiation of intracellular and extracellular compartments. This is the case for MRI voxels in clinical practice: apart from some approaches currently limited to research [15,16], ^23^Na-MRI only offers ‘total’ ^23^Na signal thus far. Indeed, measurement of absolute changes (i.e. red lines; Figure 1) rather than concentration is much closer to what MRI actually does when recording signals from single atoms. Recent evidence of higher sensitivity of ^23^Na-based analysis over standard ^1^H T_2_ MRI approaches to monitor acute ischaemic extracellular compartment changes within skeletal muscle in humans [15] supports our contentions.

## Implications of the model

In our view this model has broad and important implications.

First, relative differences and/or changes in the proportion of intracellular and extracellular compartments in a tissue affect total tissue Na^+^ and K^+^ concentrations *per se*.

Second, as long as one has valid tools for its measurement (e.g. tissue chemical analysis by flame photometry/absorption spectroscopy or non-invasive MRI), Na^+^ can be much (up to 3.5–4 times) more sensitive than water itself to detect oedema. Therefore, it is no more acceptable to claim solely for a ‘more pronounced Na^+^ – than water – accumulation’ [14] to support its water-free, hypertonic nature. In fact, when parallel changes in skin or muscle water content were carefully examined, despite sometimes lacking statistical significance due to the difference in sensitivity described above, they could often be detected [9,10,13].

Third, as an Occamian consequence of the two above, our model could justify most of the proportionally higher increase in Na^+^ moles or ^23^Na-MR signal in tissues observed thus far, without the need for it to represent a novel, water-independent phenomenon. Skin and particularly the relatively acellular, glycosaminoglycans-rich dermis, where an excess of total osmolytes has been more convincingly demonstrated [2–4,14], are possible exceptions, which could offer a depot for a Na^+^ excess storage at least in part hypertonic [17]. With this regard, a parallelism between the physiology of kidney and skin, both acting as ‘barrier’ organs, has been suggested [18]. However, we wonder how much of that Na^+^ excess is due to the accumulation of Na^+^ itself or, rather, to a relative deficit of water which went lost at the barrier at some point and left its accompanying Na^+^ behind. In line with this argument, the finalistic dominance of body water – over Na^+^ – homeostasis has been recently reappraised [19].

These speculations could possibly in part reconcile contrasting positions within the field of salt research. In particular, our work does not aim to disprove the occurrence of any hypertonic Na^+^ accumulation at all, but to highlight that the high salt tissue signal observed in ageing, uncontrolled and/or secondary hypertension, diabetes, CKD, heart failure and systemic sclerosis is likely to be (at least in a considerable part) a different phenomenon. In a sense, to the ‘hypothetical framework’ described by Bhave and Neilson [17] to explain ‘real’ water-independent Na^+^ storage, our model adds another but seemingly highly prevalent mechanism for ‘apparent’ water-independent Na^+^ storage.

Furthermore, of much broader interest to the medical community at large, our model could help address the largely unmet need to diagnose subclinical and/or local oedema, as outlined below.

## The potential for ‘isotonic’ Na^+^ excess investigation

### Systemic (subclinical) congestion

The ^23^Na-MRI data collected in different patient populations at-risk-of [6,7,9,10] or with clinically overt heart failure [6,7,9,10], together with the chemical data in our rodent model of ageing and hypertension, point to a common increase in tissue Na^+^ content and, as a consequence of the model, to subclinical congestion. We now interpret many of the aforementioned ‘at–risk’ patients as affected by subclinical (or clinically unrecognized) heart failure that tissue Na^+^ analysis could make evident.

Classic diuretic interventions (i.e. loop-diuretics) or novel approaches such as SGLT2-inhibition, which ultimately produces a natriuretic/diuretic-like effect, are both effective in reducing tissue Na^+^ [7,9]. It therefore comes as no surprise that the former is the cornerstone for heart failure treatment and the latter offered tremendous and unexpected reduction in incident heart failure hospitalizations and deaths, even when used in not-overtly congested diabetic patients [20].

### Local oedema

In addition to the cardiovascular field, appreciation of the direct link between the ^23^Na signal and oedema and of its higher sensitivity to detect oedema compared with water (Figure 1, right panel; red lines) prompts development of multiple ^23^Na-MRI applications also in the whole range of diseases where mechanical or inflammatory localized oedema is implicated, including rheumatological, immunological, oncological and neurological conditions. As an example, both global and regional differences in total ^23^Na brain signal were found in multiple sclerosis patients compared to controls [16]. The diagnostic potential, even if more sophisticated analysis of intra-extracellular Na^+^ transmembrane regulation within lesions is still limited to research, seems obvious: ^23^Na-MRI could represent a radionuclide-free, ‘endogenous’ PET for early (or late) oedema. In addition, relatively inexpensive chemical investigation in preclinical models might similarly help identifying and addressing early stages of diseases.

## Conclusions

In conclusion, we acknowledge that our model lacks the detailed insights discussed extensively in many physiological reviews by others, for example in terms of interstitial dynamics and biophysical properties [17]. However, it provides a pragmatic, deliberately simple but also clear-cut demonstration that the sole addition of an extra moiety of fluid, equal in composition to the extracellular, i.e. oedema, has a considerable impact on total tissue Na^+^ *per se*. As a result, the model offers a somewhat ‘traditional’, water-paralleled perspective on what appeared as a ‘new’ phenomenon. While our hypothesis obviously warrants further validation, we believe there is clinical utility for tissue-Na^+^ analysis beyond the physiology laboratory. Our paradigm could be useful in many clinical disciplines where oedema is a key pathological component.

## Clinical perspectives

- Tissue accumulation of sodium is a recently described phenomenon and is thought to be associated with a wide range of cardiovascular diseases. It has been suggested to be water independent but remains incompletely understood.
- Based on a simple model of the chemical (water and cations) content of tissue, we demonstrate that changes in the proportion of intracellular and extracellular compartments in a tissue affect total tissue Na^+^ and K^+^ concentrations *per se* and that sodium is intrinsically more sensitive than water itself to identify oedematous conditions.
- Our model could provide an explanation that could explain the high tissue sodium content found in different pathological conditions. More importantly, tissue sodium analysis has broad potential in the early diagnosis of subclinical and/or localized oedema.

### Supporting information

**Supplementary Material:** 

## Abbreviations

ECV%: extracellular volume fraction
MRI: magnetic resonance imaging
PET: positron emission tomography
SGLT2: sodium/glucose cotransporter 2
