# Supplementary material for: Much Ado about N…atrium: modelling tissue sodium as a highly sensitive marker of subclinical and localized oedema
Source: Clin Sci (Lond). 2018 Dec 13;132(24):2609–13. doi: 10.1042/CS20180575 (PMC6365627; doi:10.1042/CS20180575)
Supplement: Supplementary file 1 [file cs-132-cs20180575_supp1.pdf]

Much Ado About N…atrium:

Modelling Tissue Sodium As A Highly Sensitive Marker Of Subclinical And Localised Oedema

Giacomo Rossitto[1], Rhian M Touyz[1], Mark C Petrie[1], Christian Delles[1]

[1]Institute of Cardiovascular and Medical Sciences, University of Glasgow, UK

SUPPLEMENTAL MATERIAL

Corresponding author:

Giacomo Rossitto, MD
Institute of Cardiovascular and Medical Sciences
BHF Glasgow Cardiovascular Research Centre
University of Glasgow
126 University Place
Glasgow G12 8TA
e-mail: Giacomo.Rossitto@glasgow.ac.uk
Phone: (+44) 0141 330 2627

Abbreviations

$[Na^+]_i$ = intracellular Na^+ concentration (mmo/l)

$[Na^+]_e$ = extracellular Na^+ concentration (mmol/l)

$[Na^+]_T$ = total Na^+ concentration in tissue (mmol/l)

$[Na^+]_{OT}$ = total Na^+ concentration in oedematous tissue (mmol/l)

$[K^+]_i$ = intracellular K^+ concentration (mmol/l)

$[K^+]_e$ = extracellular K^+ concentration (mmol/l)

$[K^+]_T$ = total K^+ concentration in a tissue (mmol/l)

$[K^+]_{OT}$ = total Na^+ concentration in oedematous tissue (mmol/l)

V_i = volume of intracellular water solution in tissue

V_e = volume of extracellular water solution in tissue

V_T = volume of total (extracellular + intracellular) water solution in tissue

ECV% = extracellular volume fraction (%) = $100 \cdot V_e/V_T$

OE% = percentage of oedema added to a tissue

Model for expected total concentration of Na^+ and K^+ in a tissue

For any tissue, considered as a sum of two different water solutions:

Na^+ total moles = Na^+ extracellular moles + Na^+ intracellular moles

Therefore:

$[Na^+]_T \cdot V_T = [Na^+]_e \cdot V_e + [Na^+]_i \cdot V_i$

or

$[Na^+]_T = [Na^+]_e \cdot V_e/V_T + [Na^+]_i \cdot V_i/V_T$

$= [Na^+]_e \cdot ECV\%/100 + [Na^+]_i \cdot (1\text{-}ECV\%)/100$

Assuming a convenient reference volume of tissue containing 1 L of total (intracellular + extracellular) water solution, absolute Na^+ tissue content (moles) numerically coincides (≈) with $[Na^+]_T$.

Similarly, for potassium:

$[K^+]_T = [K^+]_e \cdot ECV\%/100 + [K^+]_i \cdot (1\text{-}ECV\%)/100$

For a similar reference volume of tissue as above, absolute K^+ tissue content (moles) ≈ $[K^+]_T$

The last two equations were used to generate the model for expected total concentration of Na^+ and K^+ for any tissue with 15% < ECV% < 85% in its "baseline" conditions (Figure, left panel, open symbols).

Model for expected total concentration of Na^+ and K^+ in an oedematous tissue

We simulated the effect of adding a fixed and biologically plausible moiety of oedema to tissues by adding 1%, 2.5% and 5% (OE%) of a solution equal in composition to the extracellular. The above percentages were defined as v/v in relation to the "baseline" volume of the water solution in the tissue, which equals 1 L in the aforementioned convenient reference tissue.

As per first equation above, absolute Na^+ content in the oedematous tissue is:

Na^+ total moles $_{OT}$ = (Na^+ extracellular moles + Na^+ intracellular moles)$_T$ + Na^+ moles in oedema

= (Na^+ total moles)$_T$ + Na^+ moles in oedema

For the reference tissue, it numerically corresponds to

$\approx [Na^+]_e \cdot ECV\%/100 + [Na^+]_i \cdot (1\text{-}ECV\%)/100 + [Na^+]_e \cdot OE\%/100$

Na^+ concentration in the oedematous tissue is:

$[Na^+]_{OT}$ = Na^+ total moles $_{OT}$ / total Volume

= Na^+ total moles $_{OT}$ / (Volume $_T$ + Volume $_{oedema}$)

= Na^+ total moles $_{OT}$ / (1L + 1L · OE%/100)

$\approx \{[Na^+]_e \cdot ECV\%/100 + [Na^+]_i \cdot (1\text{-}ECV\%)/100 + [Na^+]_e \cdot OE\%/100\}/(1L + 1L \cdot OE\%/100)$

Similarly, for potassium:

K^+ total moles $_{OT}$ $\approx [K^+]_e \cdot ECV\%/100 + [K^+]_i \cdot (1\text{-}ECV\%)/100 + [K^+]_e \cdot OE\%/100$

$[K^+]_{OT} \approx \{[K^+]_e \cdot ECV\%/100 + [K^+]_i \cdot (1\text{-}ECV\%)/100 + [K^+]_e \cdot OE\%/100\} / (1L + 1L \cdot OE\%/100)$

$[Na^+]_{OT}$ and $[K^+]_{OT}$ equations were used to generate the model for expected total concentration of Na^+ and K^+ in an oedematous tissue, after addition of 5% oedema (Figure, left panel, closed symbols).

Changes in Na^+, K^+ and water

Percentage changes (Δ%) for absolute Na^+ content and concentration were defined, respectively, as:

Δ% absolute Na^+ content = (Na^+ total moles $_{OT}$ - Na^+ total moles $_T$) · 100 / Na^+ total moles $_T$

Δ% Na^+ concentration = $([Na^+]_{OT} – [Na^+]_T) \cdot 100 / [Na^+]_T$

Percentage changes (Δ%) for absolute K^+ content and concentration were similarly calculated but not plotted because not informative: they showed a stable decrease, numerically close to –OE%, non-significantly affected by ECV% (*data not shown*).

Percentage changes (Δ%) for water content was assumed as equal to OE%, which in fact corresponds to the v/v Δ% of the solution, rather than solvent. Of note, this approximation would at most over-estimate the Δ% for water compared to Δ% for Na^+ and K^+.

Biological assumptions

This mathematical model is obviously affected in absolute, but not relative, terms by changes in the baseline assumptions, i.e. $[Na^+]_i$, $[Na^+]_e$, $[K^+]_i$ and $[K^+]_e$: all these concentrations are subject to multiple and tight regulations (particularly for the intracellular site), which can act differently in different individuals and/or conditions. Nevertheless, despite minimal shifts along the vertical axes, curves behave consistently across multiple intra-extra cellular simulated conditions (*data not shown*), thus strengthening the robustness of the model.

For the purpose of this paper, the figure was generated assuming:

$[Na^+]_e$ = 144 mmol/l and $[K^+]_e$ = 4.64 mmol/l (as reported in an experimental setting, for comparability (1); normal values for humans: 135-145 and 3.5-5.5, respectively(2)). $[Na^+]_I$ and $[K^+]_i$ were assumed as 10 and 140 mmol, respectively, as classically reported (2).

Supplemental references

1. Titze J, Bauer K, Schafflhuber M, Dietsch P, Lang R, Schwind KH, Luft FC, Eckardt KU, Hilgers KF. Internal sodium balance in DOCA-salt rats: a body composition study. American journal of physiology Renal physiology. 2005;289:F793-802.
2. Herring NPDJ. Levick's Introduction to Cardiovascular Physiology. Sixth ed: CRC Press Book; 2018 April 10, 2018.
